# The Effect of Mipomersen in the Management of Patients with Familial Hypercholesterolemia: A Systematic Review and Meta-Analysis of Clinical Trials

**DOI:** 10.3390/jcdd8070082

**Published:** 2021-07-20

**Authors:** Behrooz Astaneh, Nima Makhdami, Vala Astaneh, Gordon Guyatt

**Affiliations:** 1 Department of Health Research Methods, Evidence & Impact (HEI), McMaster University, Hamilton, ON L8S 4K1, Canada; guyatt@mcmaster.ca; 2 Research Institute, St. Josef’s Healthcare, Hamilton, ON L8N 4A6, Canada; nmakhdam@stjosham.on.ca; 3 Faculty of Kinesiology and Health Sciences, York University, Toronto, ON M3J 1P3, Canada; astanehvala@gmail.com; 4 Department of Medicine, McMaster University, Hamilton, ON L8S 4K1, Canada

**Keywords:** familial hypercholesterolemia, mipomersen, systematic review, meta-analysis, low-density lipoproteins

## Abstract

**Background:** Familial hypercholesterolemia (FH) lead to significant adverse effects in coronary arteries. Mipomersen is a second-generation antisense oligonucleotide that inhibits the synthesis of apolipoprotein B-100, an essential component of low density lipoprotein (LDL), and thus decreases the production of LDL. We aimed to determine the effect of mipomersen in patients with FH. **Methods:** We searched Ovid Medline, Ovid EMBASE, WHO ICTRP search portal, ISI database, the reference lists of relevant articles, and also Google Scholar to retrieve articles. All randomized controlled trials (RCTs) comparing patients with FH receiving mipomersen as an add-on and a parallel group receiving a placebo or no intervention were selected. **Results:** Five studies with more than 500 patients were included. All had low risk of bias. Pooling data showed that mipomersen probably reduces LDL compared with placebo [mean difference: −24.79, 95% CI (−30.15, −19.43)] but with a moderate level of certainty. There was a high level of evidence for injection site reactions [RR = 2.56, CI (1.47–4.44)] and a low level for increased serum alanine transaminase (ALT) > 3 times upper limit of normal (ULN) [RR = 5.19, CI (1.01–26.69)]. **Conclusion:** A moderate level of evidence in decreasing serum LDL indicates that we are uncertain if this drug provides benefit in any outcome important to patients. Although a low level of evidence for an increase in serum ALT leaves uncertainty about this adverse effect, injection site reactions in 10% or more of patients can be an important concern.

## 1. Introduction

### 1.1. Description of the Condition

Familial hypercholesterolemia (FH) is an autosomal dominant disease that is caused by various mutations in a gene located on chromosome 19 whose function is to produce receptors located in sites that include liver. The function of the receptor is catching and removing low-density lipoproteins (LDL) from plasma. Different levels of mutations in the gene will lead to different severity of disturbances in LDL removal from the body [1]. Increased level of serum LDL (hypercholesterolemia) leads to major adverse effects in the cardiovascular system, including the coronary arteries as well as other organs [2]. The most severe form of the mutation leads to a complete lack of the receptor (receptor-negative mutation).

Mutations in two other genes can cause a similar phenotype; the first one is the gene related to apolipoprotein B-100 and the second one is related to the proprotein convertase subtilisin/kexin type 9 (PCSK9) [1].

In FH, malfunction of the receptor leads to the accumulation of LDL in various parts of the body, from the cornea to tendons, skin, and vessels [3,4]. The accumulation of cholesterol in the cornea causes corneal arcus, its accumulation in tendons causes xanthoma, and in the vessels, it can cause different degrees of atherosclerosis [1].

Familial hypercholesterolemia has two types; heterozygous type, which is autosomal dominant, exhibits milder forms of the disease, and its prevalence is about one in 500. The signs and symptoms of the disease, including xanthomas and coronary artery disease, are presented in later years of life (after the second decade) compared with the homozygous type [4]. The homozygous type is less prevalent (about one in a million), and the manifestations of the disease can be presented even in the first or second decade of life by very severe involvement of the vascular system, including the heart, which might need for coronary artery bypass graft surgery [2].

### 1.2. Description of the Intervention

Different therapeutic strategies have been used so far to treat FH; from dietary intervention with low effectiveness to pharmacological treatments and even invasive procedures such as plasma apheresis. In the late stage of the disease, the only effective treatment can be liver transplantation [5]. Pharmacological treatments include many drugs such as bile acid sequestrants and statins that have been used with different efficacy but mostly with lower than expected results, especially in patients with severe FH [6,7]. So the need for an add-on drug to enhance the effect of first-line treatments is suggested. New ideas include altering the absorption of cholesterol in the brush border of the intestine [7], affecting the related receptor on the liver [8], altering the pathway of producing cholesterol, or using inhibitors of proprotein convertase subtilisin/kexin type 9 (PCSK9) [9]. Apolipoprotein B-100 is an essential component of all atherogenic lipoproteins, including LDL. Altering the pathway of producing apolipoprotein B-100 can help in decreasing the production of LDL.

### 1.3. How the Intervention Might Work

Mipomersen is a new drug proposed for drug therapy of FH [10]. It is a second-generation antisense oligonucleotide that can inhibit the synthesis of apolipo-protein B-100 and thus decrease the production of LDL and lower LDL cholesterol [11,12]. The route of injection of mipomersen is subcutaneously in a formulation with 0.9% sodium chloride, which can lead to injection site reactions [13]. 

Because mipomersen targets apoB-100 mRNA in the liver and inhibits hepatic production of triglyceride-rich very low density lipoprotein (VLDL), the drug can result in the development of hepatic steatosis accompanied by elevated plasma liver transaminases [14]. The predominant distributes of mipomersen to the liver rather than the intestine results in a functional specificity for apo B100 synthesis in the liver compared with apo B48 synthesis in the intestine [15,16]. Nevertheless, investigators have reported the intestinal side effects of mipomersen because of inhibition of intestinal chylomicron production and subsequent fat malabsorption [17].” Mipomersen does not, however, have any effect on plasma PCSK9 levels [18].

### 1.4. Why It Is Important to Do This Review

Some studies have been done on the effect of mipomersen in patients with dyslipidemia, showing various effects as well as some side effects, including increased levels of liver enzymes [19,20]. Waldman et al. and Akdim et al. evaluated the effects of mipomersen in adult patients with heterozygous FH [21,22]. Raal and colleagues evaluated mipomersen as an add-on in patients with homozygous FH aged more than 12 years [15]. In a single-center placebo-controlled clinical trial, Visser and colleagues evaluated the effect of 200 mg mipomersen injection in patients with heterozygous FH [23]. However, there is no systematic review that combines the data from existing randomized clinical trials on patients with FH. Given the lack of synthesized evidence and given the seriousness of the effects of FH, it is imperative that this review be done to examine the effectiveness and safety of mipomersen. The results of this review can help clinicians, and future decision-makers be informed about the possible use of this drug. By considering the possible benefits as well as side effects, this review will assess the role of this drug as an add-on to the current therapeutic management of patients with FH. So, the objective of this review is to answer the question of what might be the effect of subcutaneous injection of mipomersen compared with placebo or no intervention in adults or pediatric patients with FH who are already on the treatment with first-line cholesterol-lowering drugs.

## 2. Materials and Methods

### 2.1. Criteria for Selecting Studies for This Review

#### 2.1.1. Types of Studies

Randomised controlled clinical trials comparing patients (adults or pediatric) with FH receiving subcutaneous injections of mipomersen as an add-on to previous pharmacologic cholesterol-lowering interventions and a parallel group receiving a placebo or no intervention. 

#### 2.1.2. Types of Participants

We included all the above-mentioned studies in which the participants were adults or pediatric patients with FH (either homozygous or heterozygous) who were already on the treatment with cholesterol-lowering interventions.

#### 2.1.3. Types of Interventions

Subcutaneous injections of mipomersen (any dose and duration) as an add-on to previous pharmacological cholesterol-lowering interventions compared with a parallel group receiving a placebo or no intervention. We excluded the studies that used non-pharmacological interventions such as plasma apheresis instead of pharmacological ones. We also excluded trials whose parallel comparator group received active treatments.

#### 2.1.4. Types of Outcome Measures

Decreased major adverse cardiovascular events (MACE) defined as cardiovascular death, acute myocardial infarction, or unstable angina, liver toxicity, percent of the decrease in serum LDL, risk of developing injection site reaction, and risk of increased serum alkaline aminotransferase (ALT) level at least 3 times more than upper limit of normal (ULN) were considered as outcomes. 

### 2.2. Search Methods for Identification of Studies

#### 2.2.1. Electronic Searches

We searched Ovid EMBASE (from 1974 to 27th January 2020) using the search terms and combinations in Table S1, and Ovid Medline (from 1946 to 27th January 2020). We also searched for the World Health Organization (WHO) International Clinical Trials Registry Platform (ICTRP) search portal (http://apps.who.int/trialsearch/, accessed on 26 February 2019) for ongoing trials to the current date. ISI Web of Science was also searched using the related keywords. We did not limit the search to any specific language, any time period, any geographical location, or the number of centers for performing the study. 

#### 2.2.2. Searching Other Resources

We also tried to contact the authors of any abstracts presented in related conferences to reach any unpublished data. The reference lists of the articles having all the inclusion and exclusion criteria were also searched for any further published articles. To assess the grey literature, Google Scholar was also searched using the keywords: “Familial hypercholesterolemia” and “Mipomersen or ISIS 301012” and “randomized trial or clinical trial or randomized clinical trial”. The first 100 hits of the Google Scholar search were reviewed for relevant articles.

### 2.3. Data Collection and Analysis

#### 2.3.1. Selection of Studies 

The titles and abstracts of the retrieved studies were screened through the Covidence software by two independent reviewers, and the full texts of the studies that passed the screening were also assessed by the same two reviewers independently considering the inclusion criteria. Any disagreement between the reviewers was resolved through discussion.

#### 2.3.2. Data Extraction and Management

A Word sheet was prepared for extracting data from the included articles, and two reviewers independently extracted data into their own sheet for the listed outcomes after piloting the process for one study. Differences between the reviewers regarding the data were resolved by discussion. 

The gathered data were about the characteristics of the studies such as the design, the number of centers of the study, sample size, trial registration numbers, inclusion and exclusion criteria, and patients’ characteristics such as age, sex, and the number of participants, type of FH, level of serum LDL before inclusion, the dose of mipomersen and duration of studies, as well as outcomes reported, and timing of outcome assessment. We planned to contact trial authors in case of any additional data required or any missing data. 

#### 2.3.3. Assessment of Risk of Bias in Included Studies

To assess the risk of bias of all outcomes, two authors assessed the studies independently using the recommendations in the Cochrane Handbook for Systematic Reviews of Interventions, and any disagreements were resolved by consensus. The tool used for the risk of bias assessment was the RoB2 tool of the Cochran, and the following domains were checked for any bias, including the bias arising during the randomization process (sequence generation, allocation concealment), bias due to deviations from the intended intervention (blinding of participants and personnel), bias due to missing outcome data, bias in the measurement of outcome, and bias arising from selective reporting. All items and subitems were assessed using the signaling questions of the tool, which could receive the answers as “Yes”, “No”, “Probably yes”, “Probably no, and “No information”. 

#### 2.3.4. Measures of Treatment Effect

We used Review Manager 5.3 software to perform the data analysis. For dichotomous outcomes, we expressed the difference as risk ratios (RRs) with 95% confidence intervals (CIs). For serum LDL decrease as continuous data, we expressed results as mean differences (MD) in percent of changes with 95% CI.

#### 2.3.5. Unit of Analysis Issues

One randomized controlled trial (RCT) reported two subgroups of patients separately. For analysis purposes, we treated the results as coming from two different studies [24]. In studies that included intervention arms with multiple doses, for our analysis, we included only arms receiving a dose of 200 mg/week. 

#### 2.3.6. Dealing with Missing Data

We planned to contact the authors of studies with missing data. For analysis of continuous data in which SD was not reported, we calculated SD from SE or, CIs. We reported such data in the result of the analysis of the related outcome. 

#### 2.3.7. Assessment of Heterogeneity

We assessed the heterogeneity between pooled trials using a combination of visual inspection of the graphs and also considering the Chi-squared test and the I^2^ statistic. The I^2^ value was assessed as “low or unimportant” (more than 30% and below 40%), “moderate” (30% to 60%), “substantial” (50% to 90%), or “high” (75% to 100%). We also considered the qualitative evaluation of heterogeneity.

#### 2.3.8. Assessment of Reporting Biases

We planned to generate and assess funnel plots (if there were more than 10 RCTs in meta-analysis) to assess the effects of small studies. In the case of fewer than 10 RCTs in the meta-analysis, we had to rely on the comprehensiveness of the search strategy and the range of databases we used for retrieving the related articles. 

### 2.4. Data Synthesis

We pooled the results of trials using the generic inverse variance method or Mantel-Haenszel in Review Manager 5.3. We also used random-effects model. For safety analysis, studies with zero effects were excluded from the analysis.

### 2.5. Subgroup Analysis and Investigation of Heterogeneity

In case of detecting any heterogeneity, we planned to perform sensitivity analysis for studies with a high risk of bias. We planned to do subgroup analyses to determine if the magnitude of effect was related to studies at high versus the lower risk of bias. 

### 2.6. Assessment of the Certainty of the Evidence

For all mentioned outcomes, we used the GRADE approach to evaluate the certainty of evidence. We used the following minimal important difference (MID) values when interpreting the importance of differences between the groups: for injection site reaction, we considered 25% increase in the number of patients with injection site reactions in the mipomersen group than the baseline risk, for increased in serum ALT level we considered even one more patient in 100 patients with increased at least 3 times more than ULN as MID, and for the decrease in serum LDL, we considered the MID as at least 25% decrease in LDL cholesterol after injection of mipomersen. For MACE, and liver toxicity, even one more event in 100 patients compared with the placebo group was considered important. 

### 2.7. Presentation of the Results

For a better presentation of the main results, a table for “Summary of findings” was prepared using the GRADEPro software. Decisions for downgrading the certainty of evidence were mentioned in the footnotes.

## 3. Results

### 3.1. Description of Studies and Results of the Search

**Figure 1:**
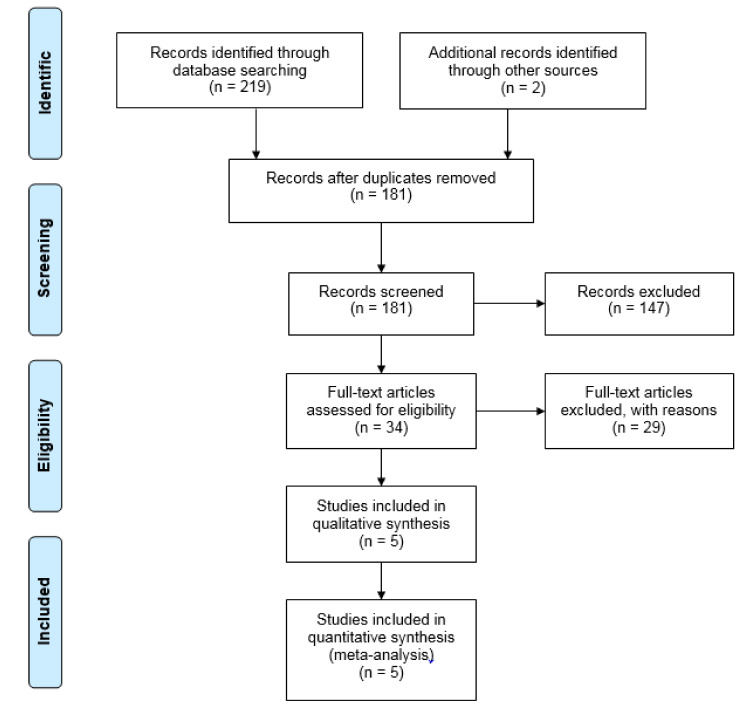
Flow diagram of selecting articles for systematical review of the effect of mipomersen on the management of familial hypercholesterolemia.

The search was done in Jan 2020. Considering the search keywords, totally 219 studies were retrieved from different databases: Embase (161), Medline (24), and Web of Science (34). Searching other sources yielded 2 more studies. Excluding 40 duplicate studies led to 181 unique reports. The titles and abstracts were screened by two reviewers, which led to 34 full-text articles. The full texts were evaluated independently by two reviewers, and the disagreements were resolved by consensus. Finally, considering the inclusion and exclusion criteria and after in-depth evaluation, five articles were selected for inclusion in the analyses. Details of the process of screening and selection of articles are shown in Figure 1 (PRISMA diagram). 

### 3.2. Included Studies

Table 1 shows the basic characteristics of the five included studies. In total 549 patients were included in all studies consisting of 369 patients in treating arms and 180 patients in the control arms. The studies were published between 2010 and 2019, and the follow-up period ranged from 6 weeks to 60 weeks. All but one study had done on adult patients with heterozygous FH [19,20,23,24]. The only one study which was done on patients with homozygous FH had mentioned in its inclusion criteria to include patients aged 12 years and more, but in practice, it could only include adults [11]. So the subgroup analysis between the adults and children that had been set up in priori could not be done. The total dose of mipomersen that had been used in almost all studies was about 200 mg per week. Akdim study had an escalating dose of 50 to 300 mg mipomersen per week in 4 arms, so the arm of 200 mg per week was selected to compare the effect on serum LDL; however, as the authors mentioned that “no evidence was found of a dose-dependent association in adverse events”, to compare injection site reactions and increased ALT, we combined all four intervention arms [19]. All studies but one were multicentre.

### 3.3. Excluded Studies

Table 2 shows the characteristics and reasons for excluding full-text studies [11,12,15,22,25,26,27,28,29,30,31,32,33,34,35,36,37,38,39,40,41,42,43,44,45,46,47,48].

### 3.4. Risk of Bias in Included Studies

**Figure 2:**
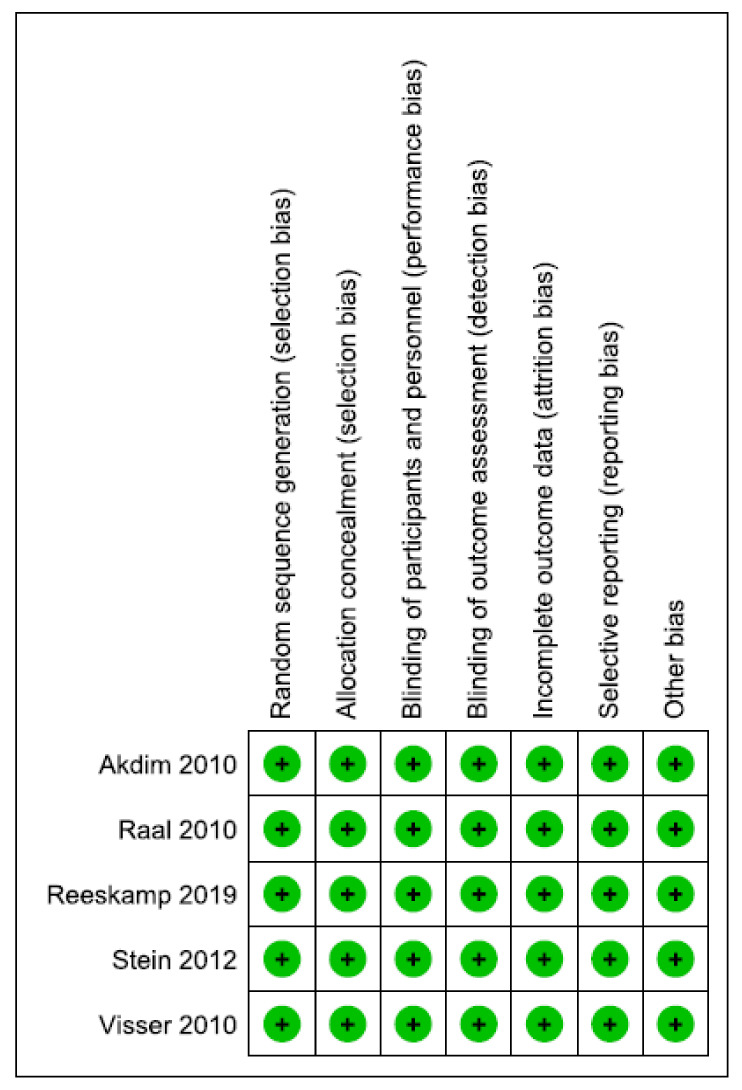
Assessment of risk of bias in each individual study.

**Figure 3:**
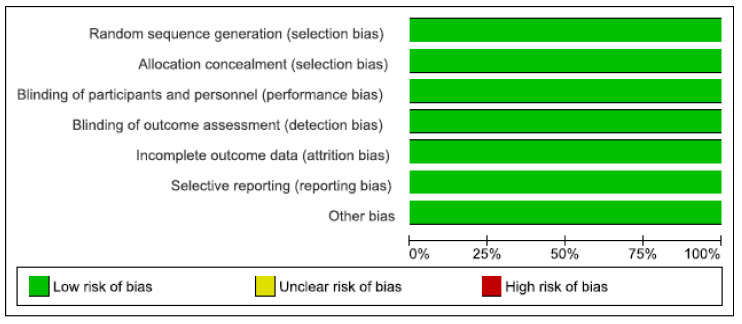
Summary of Risk of bias by domain.

We assessed risk of bias in all included studies, which showed a low risk of bias in all domains. Table 3 shows the summarised risk of bias in various domains. Figure 2 shows the assessment of risk of bias in each individual study. Figure 3 shows a summary of the risk of bias by domain. Table S2 shows detailed judgments about the risk of bias in different studies.

### 3.5. Effects of Interventions and Assessment of the Evidence

#### 3.5.1. Efficacy

##### Percent of Decrease in Serum LDL Concentration

**Figure 4:**
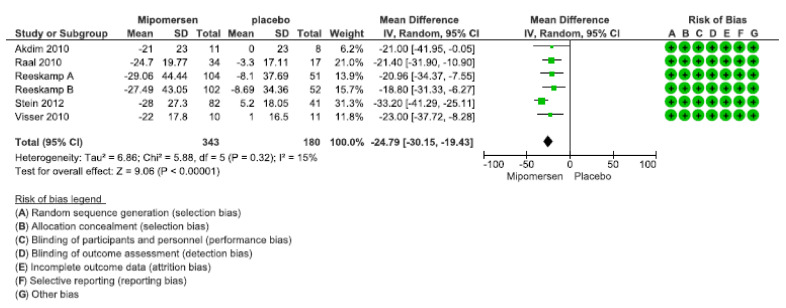
Forest plot for percent of decrease in LDL.

Five studies contributed to this analysis. One of the studies had two arms with patients with different baseline LDL to deliver either 200 mg mipomersen once weekly or 70 mg thrice weekly [24]. We separated them into studies A and B because they presented different means and SEs. Totally 523 patients provided data. Figure 4 shows the forest plot of the related meta-analysis. Two of the studies reported the changes in LDL cholesterol after receiving mipomersen or placebo as a percent of change±SD. In other studies, instead of SD, either SE or CI had been reported; for them, we had to compute the related SDs. Analysis showed a mean difference in the percent of changes in serum LDL cholesterol after using mipomersen compared with placebo as −24.79 (−30.15, −19.43). Having considered the possible side effects of mipomersen, the MID for using mipomersen was considered as at least 25% more decrease in serum LDL compared with placebo. So, we considered this during the evaluation of the level of evidence.

To assess the certainty of the evidence for this outcome, we used the GRADE approach. Risk of bias in all studies was judged to be low. To assess the heterogeneity, visual inspection of the forest plot showed that there was an overlap between the CIs of all studies. Statistical evaluation of heterogeneity shows an I^2^ = 15% and a non-significant P-value =0.32, showing an unimportant level of heterogeneity between the studies. For evaluating the imprecision, we considered that a sufficient number of patients provided data for this outcome, and all studies were in favor of using mipomersen; however as we considered the MID as at least 25% decrease in LDL after injection of mipomersen compared with placebo, then the point estimate of the mean percent of the decrease in serum LDL, and the CI around it cross the line of MID. So, we decided to decrease the level of confidence for imprecision for this outcome as −1. Comparing the PICO (Patients, Intervention, comparison, Outcome) of the included studies with ours showed no differences, so there was no need to rate down the level of evidence for indirectness. Because of the low number of studies in this analysis, we could not evaluate a funnel plot for publication bias, but the extensive search we did and the range of databases that we evaluated was comprehensive enough; so we did not rate down the level of evidence for publication bias. Finally, the level of evidence for this outcome was moderate, which means that subcutaneous injection of mipomersen probably reduces serum LDL. 

#### 3.5.2. Safety

##### Injection Site Reaction

**Figure 5:**
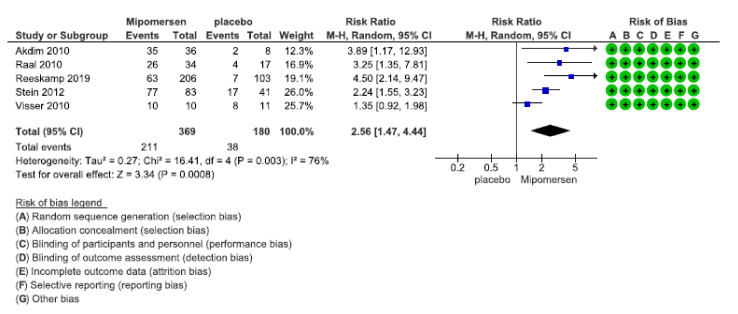
Forest plot for injection site reaction.

Five studies with 549 patients provided data for injection site reactions after mipomersen or placebo injection. Pooled analyzed data showed that the number of patients who developed injection site reactions in the mipomersen group was 2.56 (1.47–4.44) times more than patients who received placebo. Figure 5 shows the forest plot for the related analysis. To assess the certainty of the evidence, we used GRADE. Risk of bias in all studies was low. For heterogeneity, visual inspection of the forest plot shows an overlap between the CIs of almost all studies. All CIs are in favor of more injection site reactions in the mipomersen group except for a very tiny part in Visser’s study (CI: 0.92–1.98), which might because of its small sample size. Statistical evaluation of heterogeneity shows a P-value = 0.003 and I^2^ = 76%, which shows high percentages of variability due to heterogeneity. However, evaluating the patients, intervention, comparators, and the outcome showed that there were no significant differences between all the studies. Re-testing the analysis by excluding the Visser’s study showed that heterogeneity resolved (I2 = 28%) and P-value became non-significant (= 0.28), but there was not a major change in the direction of effect and also in the total RR and CI. The weight of Visser’s study was about 25% of the whole weight, although it has a small sample size. So it can be concluded that this is a relevant study that contributed to this analysis. Finally, we decided not to rate down the certainty of the evidence for heterogeneity: firstly because all point estimates suggesting the same direction and a similar magnitude of the effect. Also, CIs overlap for the most parts and, exclusion of Visser’s study, did not lead to change in the direction of effect. Secondly, it was considered that while using GRADE, statistically significant heterogeneity is only one dimension. The other dimension is the qualitative evaluation of heterogeneity. Considering the above-mentioned facts, we did not consider these studies as clinically heterogeneous. So, we did not rate down the quality of evidence just because of the quantitative measures.

For imprecision, the total events for injection site reactions were 249, which is a bit short of the optimal information size (OIS) but not too far from the 300. The baseline control rate of getting injection site reaction in patients receiving placebo is 21 in 100 patients. Having a RR = 2.56 and CI (1.47–4.44), the absolute numbers of having injection site reaction with mipomersen injections are 54 (range: 31 to 94) per 100 patients. This means 33 more patients (10 more to 73 more) in the mipomersen group developed injection site reactions compared with placebo injection. As we considered MID as 25% increase in the number of patients with injection site reactions in the mipomersen group than the baseline risk as unacceptable (25% multiply by 21 = almost 5 patients) because clinical judgment dictates that even 5 more patients with injection site reaction would be important as it can affect the tolerability of patients in receiving mipomersen injection, so looking at the RR and its CI, it is evident that even the lower end of CI (31 patients) is more than the margin of 21 + 5 = 26. With this judgment, we decided not to rate down the level of evidence for this domain because all absolute numbers of RR and CI are far more than the MID and are in favor of increasing the risk with mipomersen injection, so we judged the results to be also precise. 

Comparing the PICO of the included studies with ours showed no differences, so no need to rate down the evidence for indirectness. Because of the low number of studies in this analysis, we could not evaluate a funnel plot for publication bias, but the extensive search we did and the range of databases that we evaluated was comprehensive enough; so we did not rate down the level of evidence for publication bias. The overall certainty of the evidence for this outcome was judged to be high. So, it can be concluded that subcutaneous injection of mipomersen results in a large increase in injection site reaction. 

### 3.6. Liver Toxicity

None of the included studies reported liver toxicity, so we could not evaluate this outcome.

### 3.7. Increased Serum ALT More Than 3 Times ULN

**Figure 6:**
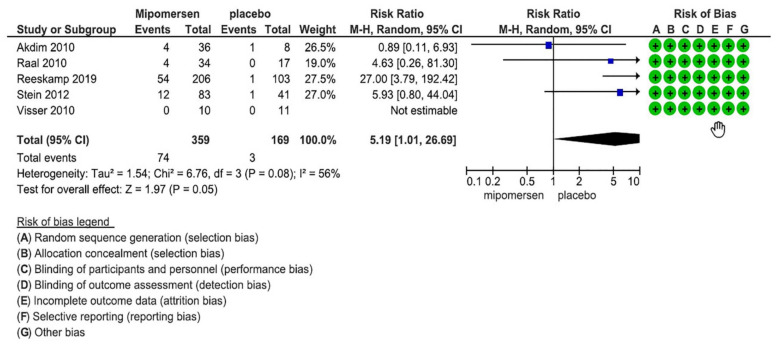
Forest plot for increased ALT.

Four studies with 528 patients provided data for this outcome. The Visser’s study did not have any event so it was excluded from the analysis [23]. Figure 6 shows the related forest plot. RR of the pooled estimate was 5.19 and CI (1.01–26.69). This shows that risk of developing an increased level of ALT more than 3 ULN is about 5 times more in patients who received mipomersen compared with placebo. To assess the certainty of the evidence, we found that risk of bias across the studies was low. Statistical evaluation of heterogeneity shows that I^2^ is 56% showing a moderate level of heterogeneity and P-value was 0.08, which is significant, but all CIs do overlap. Other than quantitatively and statistically thinking about the heterogeneity, we considered that the clinical question for this outcome is whether mipomersen can do harm by increasing ALT whose answer is yes because all the studies show degrees of harm by using mipomersen. So, clinically, the studies are not inconsistent, and we did not consider rating down the evidence for heterogeneity. 

Considering the baseline control risk of 1.8 in 100 patients (3 divided by 169) in the placebo group to develop increased ALT more than 3 ULN and considering the RR and CI, the absolute risk in the mipomersen group would be about 7 more (0–45 more) patients to develop increased ALT compared with placebo. Because an increased level of ALT for at least 3 times more than ULN can be crucial for patients’ safety, we considered even one more patient having an increase in ALT as the MID, so the CI around the point estimate crosses the MID. This fact and the low event rate (77 in total) lead to degrees of imprecision for −2 levels. Comparing our PICO with the studies’ PICO and also considering that because of few number of studies we could not do a funnel plot, but our extensive search in various databases was comprehensive, so indirectness and publication bias were not any concern in the evaluation of certainty of evidence. So, in total, we considered a low level of evidence for this outcome which means that using mipomersen may result in a large increase in the ALT, more than 3 ULN. 

### 3.8. Major Adverse Cardiac Events

**Figure 7:**
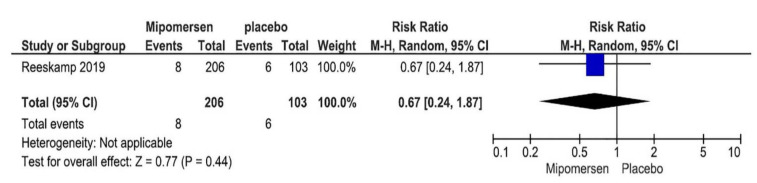
MACE plot.

Only one study evaluated MACE, so no meta-analysis was performed for this outcome [24]. In total 206 patients in the mipomersen group and 103 patients in the placebo group provided data for this outcome. Figure 7 shows the related plot. RR for this outcome was 0.67 with CI (0.24–1.87). The certainty of evidence was also assessed for MACE using the GRADE guideline. Risk of bias for all domains of this outcome was low, so the overall risk of bias was considered as low. Heterogeneity was not possible to be evaluated. Comparing the PICO of the study with our PICO did not show a significant difference. So, indirectness was not an issue for this outcome. Publication bias was not an issue because we searched all available databases and other resources to find relevant studies. However total event rate was very low. There were only 14 events in both groups, and risk ratio was 0.67 with a wide CI (0.24–1.87), which crosses the line of no difference considering the already set MID that even one more patient with MACE in the mipomersen group compared with placebo would be important. In absolute effect, as the baseline risk was developing MACE in 6 out of 100 patients in the placebo group, this wide CI means that mipomersen injection can lead to 2 fewer patients (5 fewer to 5 more patients) with MACE in the mipomersen group compared with placebo. So, this shows that the study is not precise for this outcome, and the level of evidence downgraded as −2 for this outcome and considered as low, which means that mipomersen may result in little or no difference in MACE. 

### 3.9. Summary of Findings Table 

Table 4 shows the summary of findings for different outcomes.

## 4. Discussion

### 4.1. Summary of Main Results and Certainty of the Evidence 

In patients with FH, subcutaneous injection of mipomersen probably reduces LDL compared with placebo. By pooling data yielded from 523 patients who were included in 6 RCTs, we found that the related evidence suffers from imprecision as the CI of pooled estimate crossed the MID. So, it had a moderate level of certainty. In contrast to the finding about serum LDL, we found that evidence for developing injection site reactions after mipomersen injection was high. Pooled data from five RCTs done on 549 patients showed a high level of evidence that the drug compared with placebo results in a large increase in injection site reaction. Regarding the outcome of increased in serum ALT at least more than 3 times ULN, we pooled the data of four RCTs that included 528 patients. The evidence related to this outcome suffered very severely from imprecision because of the low event rate and that CI crossed the MID. So, we had to downgrade the level of evidence to low, concluding that mipomersen may result in a large increase in the serum ALT.

We could not evaluate the effect of mipomersen on liver toxicity as no RCT had evaluated this outcome in patients with FH. There was only one RCT that reported MACE evaluated on a total of 309 patients. Very low event rate and the wide CI around the RR of comparing this outcome in both groups led us to downgrade the level of evidence because of imprecision to low and concluding that mipomersen may result in little to no difference in MACE. 

### 4.2. Overall Completeness and Applicability of Evidence 

Our review included RCTs that were done on adults. No RCT was found that compared mipomersen with placebo in children. Even the only study whose inclusion criteria was patients aged more than 12 years finally reported that the mean age of the included patients was 30 years [11]. So our reported evidence may not be applicable to children with FH. Secondly, all but one included RCTs in the meta-analysis had been done on patients with heterozygous FH. As this type of disease usually presents with milder signs and symptoms and lower serum levels of LDL, evidence reported here may not fully applicable to patients with homozygous FH. 

### 4.3. Potential Biases in the Review Process 

We tried to decrease the risk of bias in this review to the minimum by searching the related articles in all major databases. We did not limit our search to any specific language or region. All processes of article selection, screening, and assessment of the risk of bias were done by two reviewers to decrease the risk of bias. We found some abstracts presented in various congresses and tried to contact the authors for receiving the related data, but we received no response. This was not surprising because many of the research done in this field goes back to about 5–10 years ago, and the email addresses might change during this period. One of the limitations of this review might be the low number of studies done exclusively on patients with FH, which leads to a low event rate for some outcomes hence to imprecision while evaluating the level of evidence. Another limitation is that we could not evaluate the effect of mipomersen on the pediatric age group because we found no RCT done in this age group. However, the strength of our study is that this is a unique systematic review that pooled the data of RCTs exclusively done on patients with FH. 

### 4.4. Agreements and Disagreements with Other Studies or Reviews

We could not find any systematic review on RCTs done on patients with FH to compare. Fogacci et al. [49] performed a systematic review on studies that were on the effects of mipomersen on lipoproteins; however, the studies they selected had various populations with various controlled designs. They concluded that there might be favorable effects for mipomersen, but in agreement with what we found, there were also huge concerns for safety. However, in reviewing studies done on volunteers and patients with hypercholesterolemia, including those with FH, Riccota et al. reported high effectivity and safety of mipomersen either as monotherapy or combined with statin therapy, which is in contrast to what we found [37]. In reviewing 4 phase III trials, Santos et al. reported 382 patients with different types of hypercholesterolemia, including two groups with FH who received mipomersen. They reported consistent effectiveness of the drug, but they did not approach the possible side effects [39].

## 5. Conclusions

### 5.1. Implications for Practice

Moderate certainty in the evidence of decreasing serum LDL by injection of mipomersen indicates that we are uncertain if this drug provides a clinically worthwhile effect in this regard. 

Although the low level of evidence for increased serum level of ALT produces uncertainty about this side effect, the high level of evidence for injection site reactions as an important side effect of mipomersen that can affect the tolerability of patients receiving the drug can be an important concern. PCSK9 inhibitors, another add-on therapy for hypercholesterolemia, show similar injection site reactions as mipomersen [9]. Mipomersen, is not, however, associated with some adverse effects of PCSK9 inhibitors including hypersensitivity reaction and rare occurrence of leukocytoclastic vasculitis [50]. 

Lack of evidence for liver toxicity and low level of evidence for MACE may increase the ambiguities around the use of mipomersen.

### 5.2. Implications for Research

As there were no trials on children to be pooled, further research on the pediatric group with FH may affect the estimates of this review. 

Future trials should preferably include more groups of patients with homozygous FH.

## Figures and Tables

**Table 1:** Baseline characteristics of the included studies.

Study	Design of the Study	Inclusion Criteria	Duration	Groups	Patients (*n*)	Mean Age (± SD), Years	No. of Male, (%)
Reeskamp et al.	Multicentre, randomized, double-blind, placebo-controlled, parallel-group	HeFH, ≥18 years of age LDL-C ≥ 300 mg/dL or LDL-C ≥ 200 mg/dL plus documented CHD or CHD risk equivalents Maximally tolerated lipid-lowering treatment	60 weeks	Mipomersen 200 mg once weekly	67	55.2 ± 10.1	25 (37.3)
				Placebo once weekly	34	56.2 ± 10.8	13 (38.2)
				Mipomersen 70 mg thrice weekly	66	51.7 ± 12.8	27 (40.9)
				Placebo thrice weekly	33	56.1 ± 8.9	14 (42.4)
		HeFH, ≥18 years of age LDL-C ≥ 160 mg/dL or ≤200 mg/dL Documented CHD or CHD risk equivalents Maximally tolerated lipid-lowering treatment	60 weeks	Mipomersen 200 mg once weekly	37	58.5 ± 9	21 (56.8)
				Placebo once weekly	17	54.1 ± 10	9 (52.9)
				Mipomersen 70 mg thrice weekly	36	55.8 ± 9.8	21 (58.3)
				Placebo thrice weekly	19	51.5 ± 11.1	11 (57.9)
Stein et al.	Multicentre, randomized, double-blind, placebo-controlled, phase III	≥18 years of age HeFH, untreated LDL-C > 190 mg/dL Maximally tolerated statin dose, with or without other lipid-lowering treatment	26 weeks	Mipomersen 200 mg once weekly	83	56.2 ± 9.7	50 (60.2)
				Placebo once weekly	41	55.9 ± 9.3	28 (68.3)
Akdim et al.	Multicentre, randomized, double-blind, placebo-controlled, phase II	HeFH, 18–75 years of age, LDL-C ≥ 130 mg/dL, Stable conventional lipid-lowering treatment	6 weeks	Mipomersen 50 mg once weekly	8	49 ± 12	5 (62.5)
				Mipomersen 100 mg once weekly	8	53 ± 11	5 (62.5)
				Mipomersen 200 mg once weekly	11	56 ± 13	4 (36.4)
				Mipomersen 300 mg once weekly	9	47 ± 7	6 (66.7)
				Placebo	8	54 ± 10	6 (75)
Raal et al.	Multicentre, randomized, double-blind, placebo-controlled, phase III	HoFH, ≥12 years of age LDL-C ≥ 131.5 mg/dL Body weight > 40 kg Maximally tolerated lipid-lowering treatment	26 weeks	Mipomersen 200 mg once weekly	34	30.4 ± 11.5	15 (44)
				Placebo	17	33 ± 14.1	7 (41)
Visser et al.	Single-center, randomized, double-blind, placebo-controlled	HeFH, 18–75 years of age, LDL-C ≥ 100.5 mg/dL	13 weeks	Mipomersen 200 mg once weekly	10	49 ± 12	6 (60)
				Placebo	11	46 ± 1	3 (27.3)

**Table 2:** Characteristics of excluded studies.

	Study	Reason for Exclusion
1.	Stein 2010	Not a complete manuscript. Only a published abstract in the journal.
2.	Tardiff 2010 B	A duplicate article.
3.	Cromwell 2010 A	Not a complete manuscript. Only a published abstract in the journal.
4.	Patel 2010	Not an RCT
5.	Cromwell 2010 B	Not an RCT
6.	Steinhagen-Thiessen 2011	Not an RCT
7.	Visser 2011	Did not use mipomersen as an add-on therapy
8.	Visser 2009	Not a complete manuscript. Only a published abstract in the journal.
9.	Tardif 2011 A	Not a complete manuscript. Only a published abstract in the journal.
10.	Akdim 2011	Did not use mipomersen as an add-on therapy.
11.	Ricotta 2012	This was a review paper.
12.	McGowan 2012 A	Not a complete manuscript. Only a published abstract in the journal.
13.	Visser 2012	Not on patients with familial hypercholesterolemia
14.	Duell 2012	Not a complete manuscript. Only a published abstract in the journal.
15.	Mearns 2012	Not a complete manuscript. Only a published abstract in the journal.
16.	Stein 2012 B	This item has previously been included in our meta-analysis under a different title.
17.	McGowan 2012 B	Not on patients with familial hypercholesterolemia
18.	Duell 2013	Not a complete manuscript. Only a published abstract in the journal.
19.	Vogt 2013	Not an RCT
20.	Ezzahti 2013	This was a review article.
21.	Thomas 2013	Not on patients with familial hypercholesterolemia
22.	McGowan 2014	Not an RCT
23.	Duell 2014	Not an RCT
24.	Santos 2015 B	Not an RCT
25.	Santos 2015 A	Not an RCT
26.	Raal 2016	Not an RCT
27.	Ballantyne 2016	Not a complete manuscript. Only a published abstract in the journal.
28.	Duell 2016	Not an RCT
29.	Waldmann 2017	Patients had been treated with plasma apheresis instead of a pharmacologic drug.

**Table 3:** Summarized risk of bias in various domains of the included studies.

Study	Sequence Generation	Allocation Concealment	Blinding of Participants, Personnel and Outcome Assessment	Incomplete Outcome Data	Selective Outcome Reporting	Other Potential Threats to Validity
Reeskamp et al.	Low	Low	Low	Low	Low	Low
Visser et al.	Low	Low	Low	Low	Low	Low
Akdim et al.	Low	Low	Low	Low	Low	Low
Stein et al.	Low	Low	Low	Low	Low	Low
Raal et al.	Low	Low	Low	Low	Low	Low

**Table 4:** Summary of findings.

Mipomersen compared to Placebo for Familial hypercholesterolemia						
**Patient or population:** Familial hypercholesterolemia **Setting:** **Intervention:** Mipomersen **Comparison:** Placebo						
**Outcomes**	**Anticipated absolute** effects* (95% CI)		**Relative** **effect** **(95% CI)**	**No of** **participants** **(studies)**	**Certainty of** **the evidence** **(GRADE)**	**Comments**
	**Risk with** **Placebo**	**Risk with** **Mipomersen**				
Change in LDL cholesterol	The mean change in LDL cholesterol was 0 %	MD **24.79** **%** **lower** (30.15 lower to 19.43 lower)	-	523 (6 RCTs)	⨁⨁⨁◯ MODERATE ^a^	Mipomersen probably reduces LDL cholesterol. MID considered at least 25% decrease in LDL after using mipomersen
Injection site reaction	21 per 100	**54 per 100** (31 to 94)	**RR 2.56** (1.47 to 4.44)	549 (5 RCTs)	⨁⨁⨁⨁ HIGH	Mipomersen results in large increase in injection site reaction.
Increased in ALT > 3 ULN	2 per 100	**9 per 100** (2 to 47)	**RR 5.19** (1.01 to 26.69)	528 (4 RCTs)	⨁⨁◯◯ LOW ^b^	Mipomersen may result in a large increase in the serum ALT more than 3 ULN. MID considered as even one more patient with increased serum ALT more than 3 ULN after receiving mipomersen.
Major Adverse cardiac Events	6 per 100	**4 per 100** (1 to 11)	**RR 0.67** (0.24 to 1.87)	309 (1 RCT)	⨁⨁◯◯ LOW ^c^	Mipomersen may result in little to no difference in major Adverse Cardiac Events. MID considered as even one more patient with MACE after receiving mipomersen
*The risk in the intervention group (and its 95% confidence interval) is based on the assumed risk in the comparison group and the **relative effect** of the intervention (and its 95% CI). CI: Confidence interval; MD: Mean difference; RR: Risk ratio						
**GRADE Working Group grades of evidence** **High certainty:** We are very confident that the true effect lies close to that of the estimate of the effect **Moderate certainty:** We are moderately confident in the effect estimate: The true effect is likely to be close to the estimate of the effect, but there is a possibility that it is substantially different **Low certainty:** Our confidence in the effect estimate is limited: The true effect may be substantially different from the estimate of the effect **Very low certainty:** We have very little confidence in the effect estimate: The true effect is likely to be substantially different from the estimate of effect						

^a^ downgraded −1 for imprecision because point estimate and CI cross MID; ^b^ Downgraded −2 for imprecision because of low event rate and wide CI crossing the MID; **^c^** Downgraded −2 for imprecision because of low event rate and CI that crosses MID.

## Supplementary Materials

The following are available online at https://www.mdpi.com/article/10.3390/jcdd8070082/s1, Table S1: Ovid-EMBASE search, Table S2: Detailed risk of bias table of the included studies.
