# Supplementary material for: The Effect of Mipomersen in the Management of Patients with Familial Hypercholesterolemia: A Systematic Review and Meta-Analysis of Clinical Trials
Source: J Cardiovasc Dev Dis. 2021 Jul 20;8(7):82. doi: 10.3390/jcdd8070082 (PMC8304130; doi:10.3390/jcdd8070082)
Supplement: Supplementary file 1 [file jcdd-08-00082-s001.zip › jcdd-1279912-supplementary.pdf]

# Table S1: Ovid-EMBASE search

Ovid®

Wolters Kluwer

My Account Support & Training Help Feedback Logoff

Search Journals Books Multimedia My Workspace Amirsys

▼ Search History (16) View Saved

| # ▲ | Searches | Results | Type | Actions | Annotations |
|---|---|---|---|---|---|
| 1 | homozygous familial hypercholesterolemia.mp. [mp=title, abstract, heading word, drug trade name, original title, device manufacturer, drug manufacturer, device trade name, keyword, floating subheading word, candidate term word] | 922 | Advanced | Display Results \| More | |
| 2 | heterozygous familial hypercholesterolemia.mp. [mp=title, abstract, heading word, drug trade name, original title, device manufacturer, drug manufacturer, device trade name, keyword, floating subheading word, candidate term word] | 1199 | Advanced | Display Results \| More | |
| 3 | hyperlipoproteinemia type 2.mp. | 1563 | Advanced | Display Results \| More | |
| 4 | familial hypercholesterolemia.mp. [mp=title, abstract, heading word, drug trade name, original title, device manufacturer, drug manufacturer, device trade name, keyword, floating subheading word, candidate term word] | 11088 | Advanced | Display Results \| More | |
| 5 | Hyper-Low Density Lipoproteinemia.mp. [mp=title, abstract, heading word, drug trade name, original title, device manufacturer, drug manufacturer, device trade name, keyword, floating subheading word, candidate term word] | 7 | Advanced | Display Results \| More | |
| 6 | mipomersen.mp. [mp=title, abstract, heading word, drug trade name, original title, device manufacturer, drug manufacturer, device trade name, keyword, floating subheading word, candidate term word] | 728 | Advanced | Display Results \| More | |
| 7 | Kynamro.mp. [mp=title, abstract, heading word, drug trade name, original title, device manufacturer, drug manufacturer, device trade name, keyword, floating subheading word, candidate term word] | 97 | Advanced | Display Results \| More | |
| 8 | mipomersen sodium.mp. [mp=title, abstract, heading word, drug trade name, original title, device manufacturer, drug manufacturer, device trade name, keyword, floating subheading word, candidate term word] | 18 | Advanced | Display Results \| More | |
| 9 | ISIS 301012.mp. [mp=title, abstract, heading word, drug trade name, original title, device manufacturer, drug manufacturer, device trade name, keyword, floating subheading word, candidate term word] | 104 | Advanced | Display Results \| More | |
| 10 | random:.tw. or placebo:.mp. or double-blind:.tw. | 1750666 | Advanced | Display Results \| More | |
| 11 | LDL-hypercholesterolemia.mp. [mp=title, abstract, heading word, drug trade name, original title, device manufacturer, drug manufacturer, device trade name, keyword, floating subheading word, candidate term word] | 55 | Advanced | Display Results \| More | |
| 12 | ApoB synthesis inhibitor.mp. [mp=title, abstract, heading word, drug trade name, original title, device manufacturer, drug manufacturer, device trade name, keyword, floating subheading word, candidate term word] | 18 | Advanced | Display Results \| More | |
| 13 | Apolipoprotein B antisense.mp. [mp=title, abstract, heading word, drug trade name, original title, device manufacturer, drug manufacturer, device trade name, keyword, floating subheading word, candidate term word] | 16 | Advanced | Display Results \| More | |
| 14 | (or/1-5) or 11 | 12240 | Advanced | Display Results \| More | |
| 15 | (or/6-9) or 12 or 13 | 783 | Advanced | Display Results \| More | |
| 16 | 10 and 14 and 15 | 161 | Advanced | Display Results \| More | |

Contract

## Table S2: Detailed risk of bias table of the included studies

## Akdim 2010

| Bias | Authors' judgment | Support for judgment |
|---|---|---|
| **Random sequence generation (selection bias)** | Low risk | Page 1414 "A multicenter, randomized, placebo-controlled, double-blind, dose-escalation design was used for this Phase II study. The eligible participants were randomized 4:1.”<br><br>Based on the above statements, the risk of bias in this domain can be considered to be low. |
| **Allocation concealment (selection bias)** | Low risk | Page 1414: " The patients, investigators, and study staff were unaware of the treatment assignments "<br><br>This can help us conclude that the study was at low risk for selection bias. |
| **Blinding of participants and personnel (performance bias)** | Low risk | Page 1414 "A multicenter, randomized, placebo-controlled, double-blind, study."<br>Based on this statement, patients were blinded to the allocation, and therefore the risk of bias for this domain is low |
| **Blinding of outcome assessment (detection bias)** | Low risk | Page 1414 of the study: “Patients, investigators, and study staff were blinded to the treatment assignment with the exception of the pharmacist who prepared the study drug. “This randomized, placebo-controlled study was double-blinded. |
| **Incomplete outcome data (attrition bias)** | Low risk | Page 1416 of the study: "Of the 55 screened patients, 44 were enrolled in the study, at one site in The Netherlands and six sites in the United States".<br>Page 1416:” The study endpoints were analyzed on the intent-to-treat population (n44). Missing values were imputed by identifying the assessment closest to 2 weeks after the last dose of the study drug.”<br><br>The study provides a detailed explanation about all enrolled patients and their outcome in the study and also a detailed explanation about analysis of missing data. Therefore, the risk of bias for this domain is considered as low. |
| **Selective reporting (reporting bias)** | Low risk | Page 1413 of study: "This trial has been registered at Clinicaltrials.gov as NCT00281008."<br>Page 1416: “The study endpoints were analyzed on the intent-to-treat population “<br>The reported outcomes are following the study protocol that has been registered to Clinicaltrials.gov. Moreover, data were analyzed based on intention to treat analysis, therefore, it is unlikely that the result that was assessed to have been selected. The risk of bias in this domain is considered low. |
| **Other bias** | Low risk | |

**Raal 2010**

**Risk of bias table**

| **Bias** | **Authors' judgment** | **Support for judgment** |
|---|---|---|
| **Random sequence generation (selection bias)** | Low risk | Page 1000 of the study: " After a 4-week screening phase, patients were randomly assigned in a 2:1 ratio to 26 weeks of treatment with mipomersen (Isis Pharmaceuticals, Carlsbad, CA, USA) 200 mg per week (160 mg if bodyweight <50 kg) or a matching volume of placebo administered subcutaneously. Randomization was computer generated and was stratified by weight (motivated by the different doses used for patients weighing <50 kg vs ≥50 kg) in a centralized blocked randomization, which was then implemented with a computerized interactive voice response system (IVRS)"<br><br>Based on this statement from the study, patients were randomly assigned to intervention and placebo and the risk of bias is therefore considered as low. |
| **Allocation concealment (selection bias)** | Low risk | Page 1000 "The IVRS provided sites with blinded and coded medication kits to be dispensed to ensure that all clinical, medical, and pharmacy personnel, as well as the patient, remained masked to treatment allocation." |
| **Blinding of participants and personnel (performance bias)** | Low risk | Page 1000 of the study: "All clinical operations, data management, and statistical personnel also remained masked until the database was locked" Also All study personnel were blinded during the study and therefore in allocating the participants to their randomly assigned groups and the risk of bias for this domain can be concluded low for this category. |
| **Blinding of outcome assessment (detection bias)** | Low risk | Page 1000 of the study "All clinical operations, data management, and statistical personnel also remained masked until the database was locked."<br><br>The risk of bias for this domain is therefore categorized as low. |
| **Incomplete outcome data (attrition bias)** | Low risk | Page 1002 :"51 were enrolled (34 mipomersen, 17 placebo). 45 patients completed the 26-week treatment period (28 mipomersen, 17 placebo). Reasons for withdrawal from the mipomersen group were: injection- |

| | | |
|---|---|---|
| | | site reactions (n=2); rash (n=1); ALT increase (n=1); non-compliance (n=1); and consent withdrawn (n=1). Overall mean percentage compliance with study drug was 98 · 3% (SD 6 · 4; 95% CI 96–101) for patients in the mipomersen group and 99 · 8%"<br><br>The study results provide sufficient information on study participants and their outcomes and follow-ups. Therefore the risk of bias in this domain is low. |
| **Selective reporting (reporting bias)** | Low risk | Page 1001 of the study" This trial is registered with ClinicalTrials.gov, number NCT00607373."<br>Page 1001:" The differences between groups in change from baseline to the primary efficacy time point were assessed with a<br>population defined as all patients who received study drug and who had at least one lipid assessment after baseline (ie, an intention-to-treat analysis). This analysis included all patients randomly assigned in this study; thus the analysis presented is the full analysis set."<br><br>Data that produced the result were analyzed following a pre-specified protocol that has been reported in Clinicaltrials.gov. Additionally, the data for all participants were analyzed based on intention to treat. Therefore, there is a low risk that the results to be selectively reported |
| **Other bias** | Low risk | |

**Reeskamp 2019**

**Risk of bias table**

| **Bias** | **Authors' judgement** | **Support for judgement** |
|---|---|---|
| **Random sequence generation (selection bias)** | Low risk | Page 110 of study: " A multicenter, randomized, double-blind, placebo-controlled, parallel-group study was performed at 131 sites in 30 countries"<br><br>"for a study overview. Patients were then stratified by gender and use of statins and randomized at a 2:1 ratio to receive mipomersen or placebo. The total study duration consisted of a 60-week blinded treatment phase followed by a 24-week post-treatment safety follow-up period" |

| | | |
|---|---|---|
| | | Study participants were randomly and stratified based on their geographical region to either of intervention or control and the risk of bias for this domain is low. |
| **Allocation concealment (selection bias)** | Low risk | Patients, investigators, and study staff were blinded to treatment allocation and lipid data of the patients during the entire blinded treatment period" |
| **Blinding of participants and personnel (performance bias)** | Low risk | "Patients, investigators, and study staff were blinded to treatment allocation and lipid data of the patients during the entire blinded treatment period."<br>Based on this statement from page 110 of this study, patients and investigators were blinded to the randomized groups. |
| **Blinding of outcome assessment (detection bias)** | Low risk | Page 110 of the study "Patients, investigators, and study staff were blinded to treatment allocation and lipid data of the patients during the entire blinded treatment period." |
| **Incomplete outcome data (attrition bias)** | Low risk | Page 111: "A total of 309 subjects were enrolled in this study and randomized among 4 different treatment arms within severe HeFH and high-risk HeFH patients; 104 subjects received mipomersen 200 mg once weekly, 51 received placebo once weekly, 102 subjects received mipomersen 70 mg thrice weekly, and 52 subjects received placebo thrice weekly."<br><br>Based on tables 2-4 outcomes were reported for all subjects in the 4 groups. The study has provided a detailed explanation of the outcome of all participants who were enrolled in the study. Risk of bias is considered low for this domain. |
| **Selective reporting (reporting bias)** | Low risk | This clinical trial has been registered in Clinicaltrials.gov under the number of NCT00706849. Also, the study was analysed based on intention to treat. The published reports are in accordance with the predefined sets of outcomes and the risk of bias in this domain can be considered as low, given the above-mentioned facts. |
| **Other bias** | Low risk | |

**Stein 2012**

**Risk of bias table**

| Bias | Authors' judgment | Support for judgment |
|---|---|---|
| **Random sequence generation (selection bias)** | Low risk | Page 2284: "This randomized, double-blind, placebo-controlled, phase 3 trial was conducted in 26 lipid clinics in Canada and the United States" ".Eligible patients were randomized 2:1"<br>Patients are randomly assigned to either receive intervention or placebo and the risk of bias for this domain is low. |
| **Allocation concealment (selection bias)** | Low risk | "Eligible patients were randomized 2:1 to weekly subcutaneous<br>mipomersen 200 mg or placebo for 26 weeks. Patients and study personnel were blinded to treatment assignment and to lipid data."<br>Based on this statement of the page 2284 of the study and given that the study personnel was blinded to patient allocation, it can be concluded that the allocation was concealed and the risk of bias in this domain in low. |
| **Blinding of participants and personnel (performance bias)** | Low risk | Page 2284: "Patients and study personnel were blinded to treatment assignment and to lipid data."<br><br>The risk of bias in this domain is low as the patients and study personnel are all blinded to allocation. |
| **Blinding of outcome assessment (detection bias)** | Low risk | Page 2284:"Patients and study personnel were blinded to treatment assignment and to lipid data."<br>Page 2285: "Hepatic fat was measured at baseline and within 2 weeks of treatment completion or discontinuation; measurements were evaluated centrally (MR3T Bydder Laboratory, University of California, San Diego) by an assessor blinded to treatment assignment."<br><br>Outcome assessors were blinded to patient allocation. |
| **Incomplete outcome data (attrition bias)** | Low risk | Page 2286 "A total of 225 patients were screened, 124 were randomized (41 placebo, 83 mipomersen), and 114 completed treatment (41 placebo, 73 mipomersen)",<br>Nine mipomersen patients withdrew from treatment after a mean of 10.3 weeks (range, 2–17) for the following reasons: noncardiac chest pain (n 2); constipation (1); injection site reaction (n 3; 1 also had influenza-like symptoms); "<br>The study provides a detailed explanation about the outcomes of all the participants including those who did |

| | | |
|---|---|---|
| | | not complete the study due to any reason. The risk of bias assessment concludes low risk for this domain. |
| **Selective reporting (reporting bias)** | Low risk | Page 2285: "Between-group differences in LDL cholesterol change were assessed with the use of an intention-to-treat analysis, incorporating all patients who received study medication who had at least 1 postbaseline lipid assessment."<br>This clinical trial has been registered in Clinicaltrials.gov (NCT00706849) (page 2283) with a predefined set of outcomes that have been reported in this RCT. Moreover, results were analyzed on an intention to treat basis as described on page 2285 of the study. The published reports are in accordance with the predefined sets of outcomes and reporting bias is therefore considered low. |
| **Other bias** | Low risk | |

**Visser 2010**

**Risk of bias table**

| **Bias** | **Authors' judgment** | **Support for judgment** |
|---|---|---|
| **Random sequence generation (selection bias)** | Low risk | Page 1058: "Patients were randomized at a 1:1 ratio (active: placebo)."<br>The risk of bias in this domain is therefore low |
| **Allocation concealment (selection bias)** | Low risk | "Patients, investigators, and study staff were blinded to the treatment assignment with the exception of the pharmacist who<br>prepared the study drug"<br><br>Based on the above-mentioned paragraph, the allocation has been performed by blinded study personnel; thus, there is a low risk for selection bias in this category. |
| **Blinding of participants and personnel (performance bias)** | Low risk | Page 1058: "Patients, investigators, and study staff were blinded to the treatment assignment with the exception of the pharmacist who prepared the study drug."<br>Given the above statement, the patients and study personnel were blinded. |
| **Blinding of outcome assessment (detection bias)** | Low risk | Outcome assessors were blinded to the patient's allocation based on this statement from the page 1058 of the study: "Patients, investigators, and study staff were blinded to the treatment assignment with the exception of the pharmacist who prepared the study drug." |

| | | |
|---|---|---|
| **Incomplete outcome data (attrition bias)** | Low risk | “Page 1059:” Missing lipid parameter and liver triglyceride content values at day 99 were replaced by the last observation carried forward method (LOCF)”<br><br>Page 1059: "Twenty-one subjects with heterozygous FH and fulfilling all other entry criteria were randomly assigned to either mipomersen or placebo treatment", "Twenty subjects completed the study protocol. One mipomersen treated subject discontinued treatment after the first dose due to flu-like symptoms."<br>Adequate information is provided on the study participants and their outcomes from the study. Also, the study provides information on how the missing data form participants were analyzed. The risk of bias in this domain can be considered low. |
| **Selective reporting (reporting bias)** | Low risk | Page 1057 footnote: “This study was funded by ISIS Pharmaceuticals, Inc., Carlsbad, CA This study is registered at clinicaltrials.gov as NCT00362180”<br><br>"Page 1059: “Study endpoints were analyzed on the intention-to-treat population, consisting of all 21 subjects randomized as well as on the 6 FHBL positive controls."<br>This RCT has been registered in Clinical trials.gov and the predefined outcomes are reported at the study results section. Moreover, the study endpoints were analyzed on the intention to treat population. It can be concluded that the risk of selective reporting is low. |
| **Other bias** | Low risk | |
